# PATTERNS OF ANTIHYPERTENSIVES CHANGE AND MORTALITY IN VA NURSING HOME RESIDENTS

**DOI:** 10.1093/geroni/igad104.1851

**Published:** 2023-12-21

**Authors:** Xiaojuan Liu, Michelle Odden

**Affiliations:** Stanford University, Stanford, California, United States; Stanford University, Stanford, California, United States

## Abstract

Antihypertensive treatment is common in nursing home residents, yet data on the frequency and pattens of treatment changes in this group are lacking. We described the patterns of antihypertensive treatment changes and examined the mortality rate in 24870 Veterans Affairs (VA) nursing home residents ≥65 years with long-term stays (≥180 days) from 2006-2019. The number and dose of antihypertensive use was averaged weekly, and two change events were defined: deprescribing (reduced number of antihypertensives or dose of ≥30% compared to previous week) and intensification (opposite to deprescribing). More than 91% of residents were prescribed antihypertensive and 75% experienced at least one event during their stay. Death occurred in 7881 (32%) residents. We categorized residents into 10 distinct pattern groups: no change (27%), 1 deprescribing event (11%), 1 intensification event (10%), multiple deprescribing events (5%), multiple intensification events (7%), 1 deprescribing followed by 1 intensification (3%), 1 intensification followed by 1 deprescribing (4%), 3 changes with mixed deprescribing/intensification (7%), >3 changes with mixed deprescribing/intensification (10%), and no antihypertensive use (15%). We found treatment changes were more frequent in residents with better mobility and cognitive function. Residents who experienced deprescribing events had higher mortality than those experienced intensification events. The highest mortality was for those without medication (incidence = 344/1000 person-year) and this group also had the worst cognitive function. These findings indicate that complex patterns of medication changes exist in VA nursing home residents. A better understanding of the consequence of antihypertensive treatment changes could inform clinical practice in this population.

